# Characterizing the gut (*Gallus gallus*) microbiota following the consumption of an iron biofortified Rwandan cream seeded carioca (*Phaseolus Vulgaris* L.) bean-based diet

**DOI:** 10.1371/journal.pone.0182431

**Published:** 2017-08-10

**Authors:** Spenser Reed, Hadar Neuman, Raymond P. Glahn, Omry Koren, Elad Tako

**Affiliations:** 1 USDA-ARS Robert W. Holley Center for Agriculture & Health, Cornell University, Ithaca, NY, United States of America; 2 Division of Nutritional Sciences, Cornell University, Ithaca, NY, United States of America; 3 Faculty of Medicine, Bar-Ilan University, Safed, Israel; Defense Threat Reduction Agency, UNITED STATES

## Abstract

Biofortification is a plant breeding method that introduces increased concentrations of minerals in staple food crops (e.g., legumes, cereal grains), and has shown success in alleviating insufficient Fe intake in various human populations. Unlike other strategies utilized to alleviate Fe deficiency, studies of the gut microbiota in the context of Fe biofortification have not yet been reported, although the consumption of Fe biofortified staple food crops has increased significantly over time. Hence, in this study, we performed a 6-week feeding trial in *Gallus gallus* (*n* = 14), aimed to investigate the alterations in the gut microbiome following administration of an Fe biofortified bean-based diet (biofortified, BFe) versus a bean based diet with poorly-bioavailable Fe (standard, SFe). Cream seeded carioca bean based diets were designed in an identical fashion to those used in a recent human clinical trial of Fe biofortified beans in Rwanda. We hypothesized that the different dietary Fe contents in the beans based diets will alter the composition and function of the intestinal microbiome. The primary outcomes were changes in the gut microbiome composition and function analyzed by 16S rRNA gene sequencing. We observed no significant changes in phylogenetic diversity between groups. There were significant differences in the composition of the microbiota between groups, with the BFe group harboring fewer taxa participating in bacterial Fe uptake, increased abundance of bacteria involved in phenolic catabolism, and increased abundance of beneficial butyrate-producing bacteria. Additionally, depletion of key bacterial pathways responsible for bacterial viability and Fe uptake suggest that improvements in Fe bioavailability, in addition to increases in Fe-polyphenol and Fe-phytate complexes due to biofortification, led to decreased concentrations of cecal Fe available for bacterial utilization. Our findings demonstrate that Fe biofortification may improve Fe status without negatively altering the structure and function of the gut microbiota, as is observed with other nutritional methods of Fe supplementation. These results may be used to further improve the efficacy and safety of future biofortification efforts in eradicating global Fe deficiency.

## Introduction

Iron (Fe) deficiency is the most prevalent dietary micronutrient insufficiency worldwide [1]. Significant morbidity and mortality are associated with insufficient Fe intake [2], and cognitive and physical impairment in vulnerable populations (e.g., children, pregnant women) are especially common [3]. Fe deficiency is particularly prevalent in low-income countries due to a lack of animal product consumption, in addition to dietary reliance on grains and legumes that contain significant amounts of phytic acid and polyphenolic compounds, inhibitors of Fe absorption [4]. In Rwanda alone, >50% of children suffer from Fe-deficiency anemia [5]. The common bean (*Phaseolus vulgaris* L.) is a nutritious staple food crop that is widely consumed by target populations in Central and East Africa, as well as in Latin America [6,7]. Insufficient Fe intake, poor Fe absorption, and/or additional dietary Fe requirements that tax physiological needs (e.g., pregnancy) are central to this issue [8]. To alleviate Fe deficiency, biofortification has been proposed to complement existing efforts [9]. Biofortification uses both conventional plant breeding and genetic modification to increase concentrations of minerals in staple food crops, and has become an effective tool to address micronutrient deficiencies, especially that of Fe, in many at-risk populations [10]. Indeed, our group recently published evidence in this journal for significant yet limited improvement in Fe status provided by Rwandan cream seeded carioca (*Phaseolus Vulgaris* L.) bean-based diet [11], the same bean-based diet used in the current study.

The gut microbiota is known to play a prominent role in host nutritional status, including the modulation of saccharide uptake [12], influencing energy balance [13], and *de novo* biosynthesis of various vitamins and minerals [14]. Fe is an essential nutrient for many microbes coexisting within the intestinal environment [15,16], and it has been recently demonstrated that the gut microbiota has the potential to influence Fe uptake and storage by modulating epithelial Fe transport proteins [17]. Studies in both humans and animals have reported changes in the gut microbial composition due to Fe supplementation, including increases in *Bacteroides* spp. and members of the Enterobacteriaceae [18,19], decreases in bifidobacteria and Lactobacilli [18, 20–22], and an expansion of opportunistic pathogens such as *Salmonella*, *Escherichia coli*, and *Clostridium difficile* [19,23]. Recent studies using animal models of dietary Fe deficiency have shown decreased levels of *Bacteroides* spp. and *Roseburia* spp./*Eubacterium rectale* and increased levels of lactobacilli and Enterobacteriaceae [24,25]. It is clear that dietary components, such as the Fe, are major factors in influencing the host gut microbiota composition and metabolism [26].

In addition, there is a large body of evidence that now suggests that poorly bioavailable Fe can stimulate the growth and virulence of pathogenic microbes in the intestinal milieu, and that host Fe status influences defense against these pathogens [27]. As such, Fe availability to microorganisms is generally limited by the host to prevent dysbiosis and outgrowth of these taxa [28]. Although Fe status modulates the gut microbiota and, thus, host health [29], there have been no reports evaluating the gut microbiota in subjects consuming Fe biofortified diets. As the consumption of Fe biofortified diets increases due to the increasing implementation of biofortification strategies, understanding the risk:benefit ratio from the perspective of the gut microbiota remains important if we are to further improve the nutritional outcomes provided by biofortification.

Therefore, the present study examined the compositional and functional changes to the gut microbiota in broiler chickens fed a relatively Fe bioavailable diet (biofortified, BFe) versus a Fe poorly-bioavailable diet (standard, SFe). Biofortified bean based diets were designed in identical fashion to those used in a recent human efficacy trial of biofortified cream seeded carioca beans (*Phaseolus vulgaris* L.) in Rwanda [7]. A panel of physiological markers were measured weekly to monitor the level of Fe deficiency, and gene expression of a variety of Fe-related proteins was quantified from the duodenum at the study conclusion [11]. 16S rRNA gene sequencing was used to analyze the microbial changes in the cecal contents.

## Methods

### Ethics statement

All animal protocols were approved by the Cornell University Institutional Animal Care and Use committee (protocol name: *Intestinal uptake of Fe and Zn in the duodenum of broiler chicken*: *extent*, *frequency and nutritional implications*; protocol number: 2007–0129).

### Animals, diets, and study design

Upon hatching, chicks (Cornish cross) were randomly allocated into two treatment groups on the basis of body weight and gender (aimed to ensure equal distribution and minimize bias between groups, *n* = 14): 1. Fe Biofortified (BFe): 34.6% biofortified cream seeded carioca bean based diet (48.7 ± 1.50 μg Fe/g), and 2. Fe Standard (SFe): 34.6% biofortified cream seeded carioca bean based diet (33.7 ± 0.80 μg Fe/g). Details of the diets are shown in S1 Table. At study conclusion (day 42), birds were euthanized (CO_2_ exposure). The digestive tracts (colon and small intestine) and liver were rapidly removed and frozen as was previously described [30].

### Biochemical analysis and hemoglobin (Hb) determination

Blood samples were collected weekly from the wing vein (*n* = 14, 100μL) using micro-hematocrit heparinized capillary tubes (*Fisher Scientific*, Pittsburgh, PA). Samples were collected in the morning following an 8 h overnight fast. Weekly blood Hb concentrations were determined spectrophotometrically using the cyanmethemoglobin method (H7506-STD, *Pointe Scientific Inc*., Canton, MI) following the kit manufacturer’s instructions. Fe bioavailability was calculated as hemoglobin maintenance efficiency (HME) as previously reported [11].

### Isolation of total RNA

Total RNA was extracted from 30 mg of duodenal (proximal duodenum, *n* = 14) and liver tissues (*n* = 14) as described in S1 Methods.

### Gene expression analysis

As previously described [31], PCR was carried out with primers chosen from the fragments of chicken duodenal tissues [DMT–1 gene (GeneBank database; GI 206597489) (forward: 5’-AGC CGT TCA CCA CTT ATT TCG-3’; reverse: 5’-GGT CCA AAT AGG CGA TGC TC-3’), DcytB gene (GI 20380692) (forward: 5’-GGC CGT GTT TGA GAA CCA CAA TGT T-3’; reverse: 5’-CGT TTG CAA TCA CGT TTC CAA AGA T-3’) and Ferroportin gene (GI 61098365) (forward: 5’-GAT GCA TTC TGA ACA ACC AAG GA’; reverse: 5’-GGA GAC TGG GTG GAC AAG AAC TC-3’). Ribosomal 18S was used to normalize the results (GI 7262899) (forward: 5’- CGA TGC TCT TAA CTG AGT-3’; reverse: 5’-CAG CTT TGC AAC CAT ACT C-3’)]. All PCR products were separated by electrophoresis on 2% agarose gel stained with ethidium bromide, and quantified using the Quantity One 1-D analysis software (*Bio-Rad*, Hercules, CA).

### 16S rRNA PCR amplification and sequencing

Microbial genomic DNA was extracted from cecal samples as described in S1 Methods.

### 16S rRNA gene sequence analysis

16S rRNA gene sequence analysis was performed as described in S1 Methods.

### Statistical analyses

All values are reported as mean±SEM. One-way ANOVA was performed to identify significant differences between the means of the experimental groups of birds, unless otherwise stated. The Kruskal–Wallis test was used to compare the relative abundance of distinct taxonomic units. Significant *P*-values (*P* < 0.05) associated with microbial clades and functions identified by LEfSe were corrected for multiple comparisons using the Benjamini and Hochberg false discovery rate (FDR) correction [32]. Statistical tests were carried out using SAS version 9.3 (*SAS Institute*, Cary, NC, USA).

## Results

### Increased Fe status and dietary Fe bioavailability in the BFe group

The BFe diet had significantly increased concentration of three dietary components: Fe (48.7μg/g±1.50 versus 33.7μg/g±0.80, respectively, *P* < 0.05), polyphenol compounds (quercetin 3-glucoside and kaempferol 3-glucoside, *P* < 0.05) and phytic acid (13793μg/g±1172 versus 10605μg/g±742, respectively, *P* < 0.05). S1 and S2 Tables provide an expanded view of the diets and the differences in polyphenol concentrations between them, respectively. In terms of Fe bioavailability, our previous *in vitro* studies using ferritin formation as a proxy for bioaccessibility demonstrated that the BFe diet contained greater amounts (*P* < 0.05) of bioavailable Fe than did the SFe diet [11].

**Fig 1:**
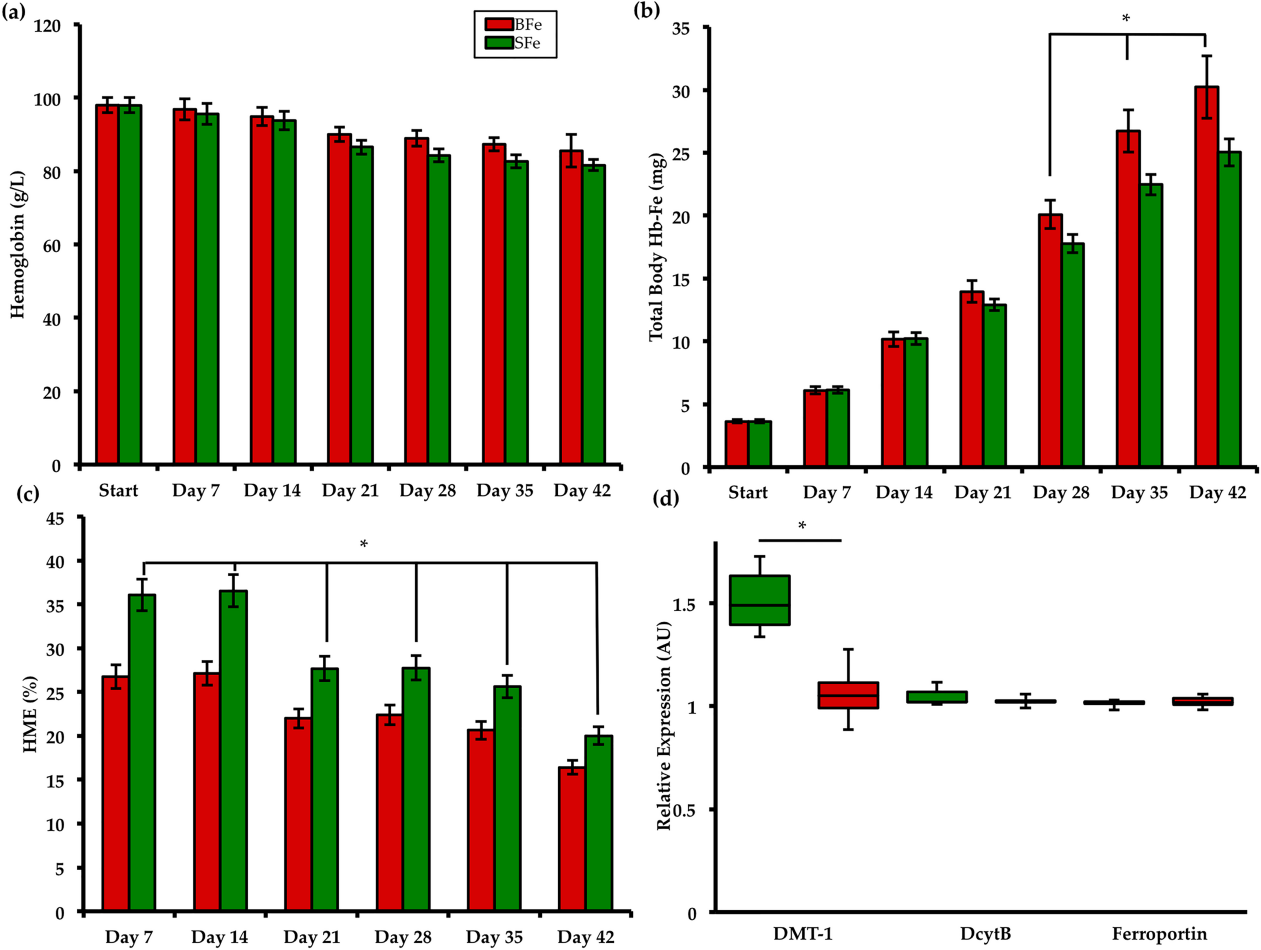
Measured Fe status parameters assessed during the study [11]. (a) Blood Hb concentration; (b) Total body Hb-Fe; (c) HME; (d) duodenal mRNA expression of Fe-related proteins collected at the end of the study (day 42).

Fig 1A–1C presents the measured hematological parameters of the two study groups [11]. In order to provide context and to demonstrate differences in Fe status between treatment groups, we used a panel of Fe status biomarkers − including standard hematological assays as well as gene expression of key Fe related proteins. Results presented in Fig 1 were adapted from our recent publication [11]. As from day 21 of the study, body weights were consistently higher in the BFe versus the SFe group (*P* < 0.05). Total body Hb−Fe (index of Fe absorption) was higher in the BFe group from day 28 until study conclusion (*P* < 0.05), while hemoglobin maintenance efficiency (HME, index of dietary Fe bioavailability) was consistently elevated in the SFe group from day 0 (*P* < 0.05). Gene expression results indicate that, relative to 18S rRNA, duodenal DMT-1 expressionwas elevated in the SFe group (*P* < 0.05). As we, and others have previously published, increased DMT-1 suggests a mechanism to compensate for the relatively low dietary Fe bioavailability in the SFe group. In addition, significantly greater hepatic Fe concentration was measured in BFe group compared to the SFe group. Altogether, the results of these Fe status parameters show that, by the end of the study, animals in the SFe were mildly Fe deficient yet non-anemic, whereas birds in the BFe group had an improved Fe status [11].

### Bacterial diversity of the gut microbiota is not significantly altered by the biofortified diet

Cecal samples from the BFe and SFe treatment groups were harvested and used for bacterial DNA extraction and sequencing of the V4 hypervariable region in the 16S rRNA gene. The cecum represents the primary site of bacterial fermentation in *Gallus gallus*, with highly diverse and abundant microbiota [33]. As in the human gut [34], Firmicutes and Bacteroidetes were by far the dominant bacterial phyla in the *Gallus gallus* cecum, accounting for > 85% of all sequences [35].

**Fig 2:**
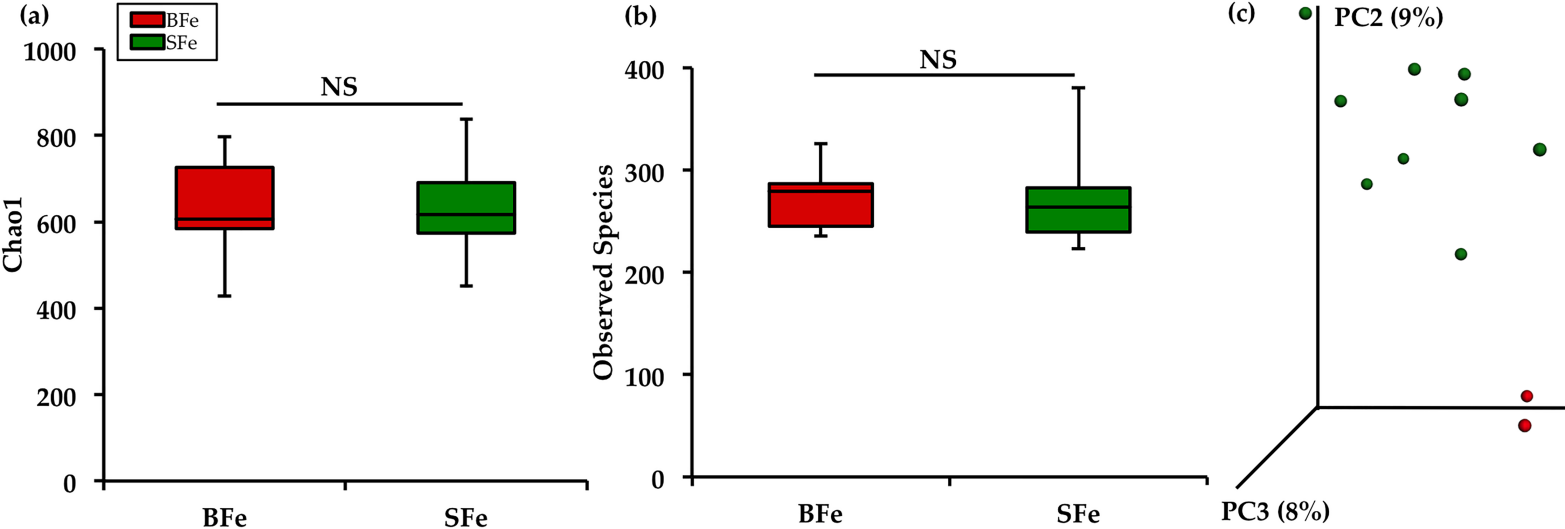
Microbial diversity of the cecal microbiome. (a) Measures of α-diversity using the Chao1 Index; (b) Total number of observed species; (c) Measure of β-diversity using unweighted UniFrac distances separated by the first three principal components (PC). Each dot represents one animal, and the colors represent the different treatment groups.

The diversity of the cecal microbiota between the BFe and SFe groups was assessed initially through measures of α–diversity and β–diversity. The Chao1 index and observed species richness were used to assess α–diversity (Fig 2A and 2B). Neither of these measures showed significant differences between the SFe and BFe groups (*P* > 0.05). We utilized unweighted UniFrac distances as a measure of β-diversity to assess the effect of the biofortified diet on between-individual variation in bacterial community composition (Fig 2C). Principal coordinate analysis (PCA) showed clear clustering of the SFe and BFe groups, suggesting that samples were more similar to other samples within each of the groups, as opposed to samples of the other group. However, distances of β-diversity within each group did not appear significantly different, demonstrating that there is similar diversity within each group (*P* > 0.05).

### Composition of the gut microbiota under Fe biofortification conditions

**Fig 3:**
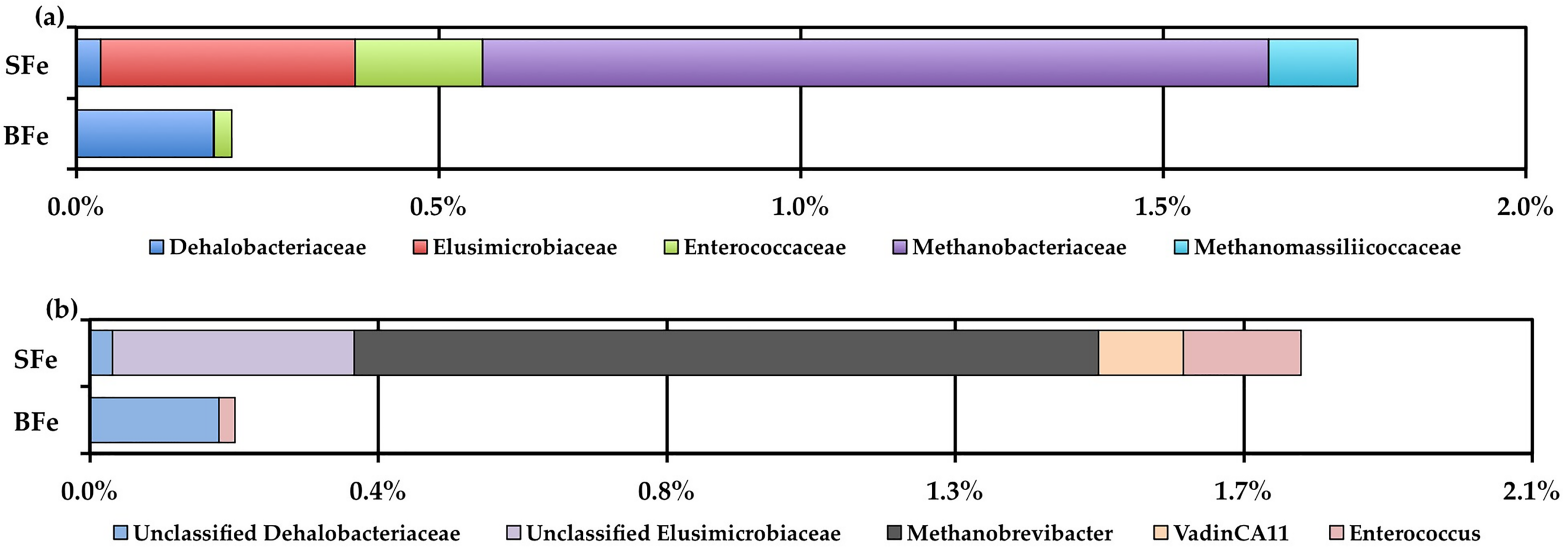
Family and genus level cecal microbiota shifts between the BFe and SFe treatment groups. (a) Family level changes in the BFe and SFe groups as measured at the end of the study (day 42); (b) Genus level changes in the BFe and SFe groups as measured at the end of the study (day 42).

We next performed a taxonomy based analysis of the cecal microbiota (Fig 3). 16S rRNA gene sequencing revealed that the vast majority of sequences in both the SFe and BFe groups were assigned to three bacterial phyla (Firmicutes, Bacteroidetes, and Proteobacteria), whereas sequences of representatives of Euryarchaeota (domain Archaea), Elusimicrobia, Cyanobacteria, Verrucomicrobia, Tenericutes, Lentisphaerae, Fusobacteria, and Actinobacteria were also identified, but in much lower abundance. The difference in abundance between the three dominant phyla, Firmicutes, Bacteroidetes, and Proteobacteria, was not significant between the BFe and SFe groups (*P* = 0.247, *P* = 0.106, *P* = 0.396, respectively). Two phyla (<1% relative abundance), Elusimicrobioa and Euryarchaeota (domain Archaea), were found to be significantly decreased in the BFe group compared to the SFe group (*P* = 0.002 and *P* = 0.001, respectively). Interestingly, no sequences of Euryarchaeota (domain Archaea) were found in the BFe group. At the family level, Elusimicrobiaceae, Methanobacteriaceae (domain Archaea), and Methanomassiliicoccaceae (domain Archaea) were significantly lower (*P* = 0.010, *P* = 0.020, *P* = 0.020, respectively), and Dehalobacteriaceae and Enterococcaceae were significantly elevated (*P* = 0.004, *P* = 0.020, respectively in the BFe group (Fig 3A). At the genus level, we detected a significantly greater abundance of unclassified *Dehalobacteriaceae* (*P* = 0.006), and significantly lower abundances of unclassified *Elusimicrobiaceae*, *Methanobrevibacter*, *vadinCA11*, and *Enterococcus* in the BFe group compared to the SFe group (*P* = 0.017, *P* = 0.038, *P* = 0.038, and *P* = 0.038, respectively, Fig 3B). It is interesting to note that the BFe group harbored no identifiable sequences of either *Methanobrevibacter* or *vadinCA11*. At the operational taxonomic unit (OTU) level, we identified one OTU belonging to the Clostridiales order, denovo2964, that was significantly enriched in the BFe group (*P* = 1.8 x 10^−5^). We were unable to further identify this bacterium.

### Discriminating the gut microbiota of the BFe and SFe groups

**Fig 4:**
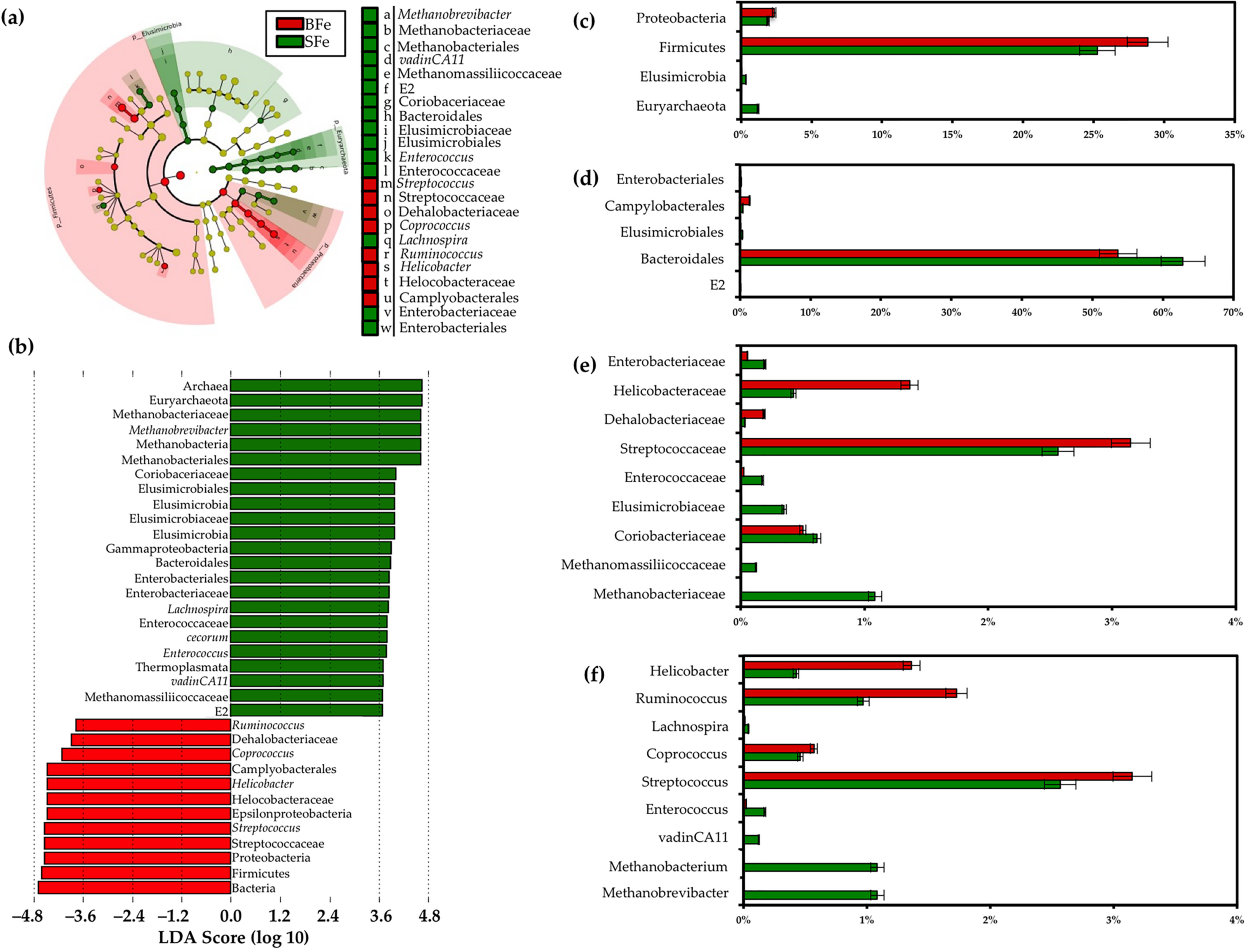
LEfSe method identifying the OTUs with the greatest differences in abundance in the BFe and SFe groups. (a) Taxonomic cladogram obtained using LEfSe analysis of the 16S rRNA sequences. Treatment groups are indicated by the different colors; (b) Computed LDA scores of the relative abundance difference between the BFe and SFe groups. Comparison of the relative abundance at the (c) phylum; (d) order; (e) family; and (f) genus levels in the BFe and SFe groups.

Examining the clade abundances at all taxonomic levels, we used the linear discriminant analysis effect size (LEfSe) method [36] to identify significant bacterial biomarkers that can discriminate the cecal microbiota of the BFe and SFe groups. Fig 4A and 4B present the differences in abundance between groups at the various taxonomic levels, with their respective LDA scores. Fig 4C–4F present the relative abundances of the sequences that were significantly different as classified by LefSe analysis.

We observed a general taxonomic delineation between the SFe and BFe groups, whereby the methanogenic Archaea predominated in the SFe group, while short chain fatty acid (SCFA)-producing Bacteria were enriched in the BFe group. At the phylum level, the Proteobacteria and Firmicutes were more abundant in the BFe group relative to that of the SFe group, whereas Elusimicrobiota and Euryarchaeota were less abundant (*P* < 0.05, Fig 4C). At the order level, the proportions of Campylobacterales were increased in the BFe group relative to that of the SFe group, whereas the proportions of Enterobacteriales, Elusimicrobiales, Bacteroidales, and E2 were decreased (*P* < 0.05, Fig 4D). At the family level, members of the Helicobacteraceae, Dehalobacteriaceae, and Streptococcaceae were enriched in the BFe group compared to the SFe group, whereas Enterobacteriaceae, Enterococcaceae, Elusimicrobiaceae, Coriobacteriaceae, Methanomassiliicoccaceae, and Methanobacteriaceae were decreased (P < 0.05, Fig 4E). At the genus level, the *Helicobacter*, *Ruminococcus*, *Coprococcus*, and *Streptococcus* were more abundant in the BFe group compared to the SFe group, whereas *Lachnospira*, *Enterococcus*, *vadinCA11*, *Methanobacterium*, and *Methanobrevibacter* were decreased. LefSe analysis also revealed several OTUs that were differentially-enriched in the BFe group compared to the SFe group; *Faecalibacterium prausnitzii* (LDA score = 5.2, *P* = 4.7 x 10^−4^), *Barnesiella viscericola* (LDA score = 4.4, *P* = 4.3 x 10^−2^), *Enterococcus cecorum* (LDA score = 4.4, 8.8 x 10^−2^), and *vadinCA11* (a novel methanogen belonging to Euryarchaeota [37], LDA score = 4.1, 9.3 x 10^−2^). In addition, an unclassified OTU belonging to *Dehalobacteriaceae* was found to be significantly enriched in the BFe group compared to the SFe group (*P* = 0.02), although this OTU was not identified by LefSe as differentially-enriched between groups.

### The metagenome of the cecal microbiota under Fe biofortified conditions

**Fig 5:**
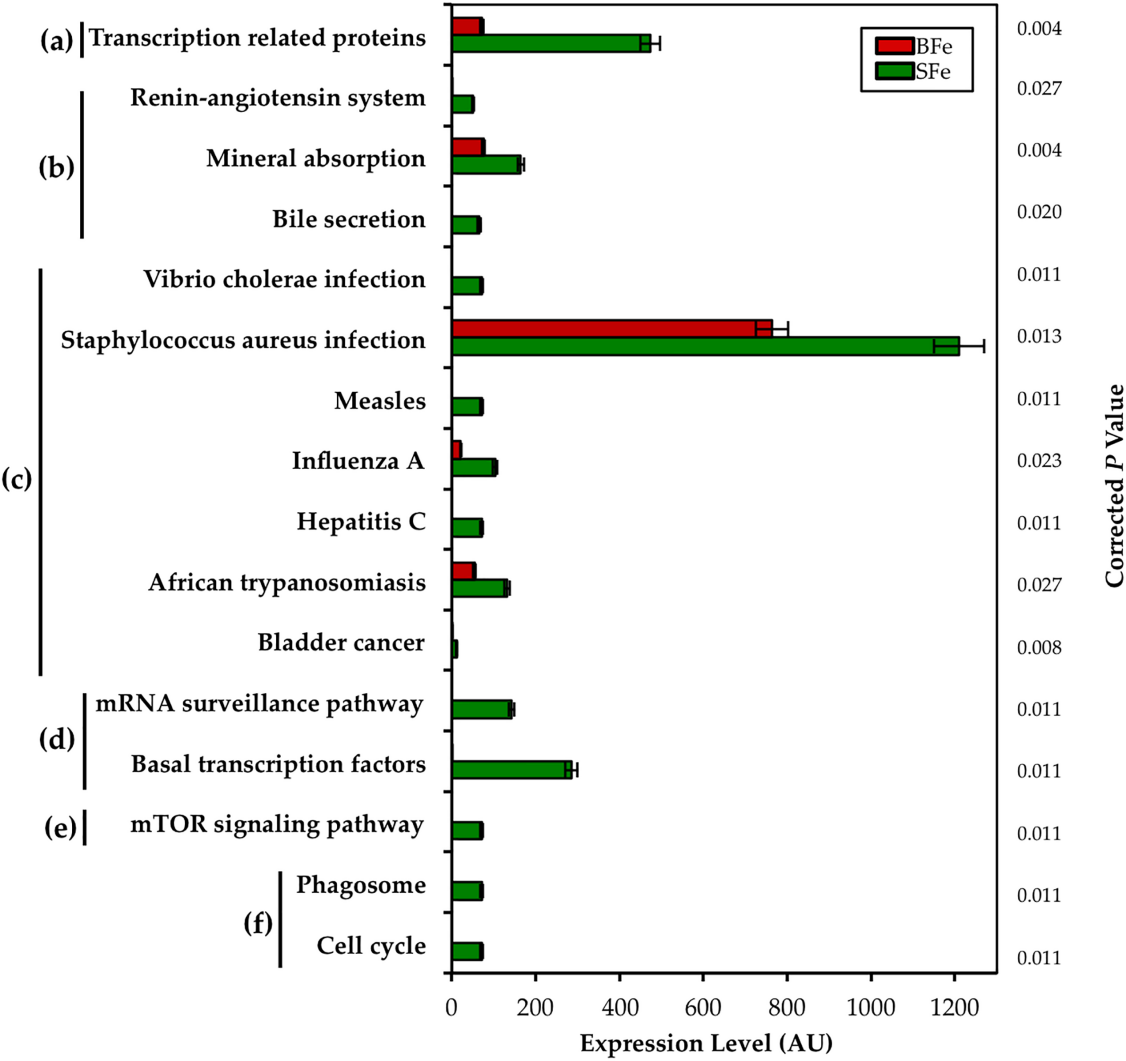
Observed alterations in the metabolic capacity of the cecal microbiota in the BFe group compared to the SFe group. **Relative abundance of differentially–enriched KEGG microbial metabolic pathways in cecal microbiota, including** a) Unclassified; b) Organismal Systems; c) Human Diseases; d) Genetic Information Processing; e) Environmental Information Processing; and f) Cellular Processes. Treatment groups are indicated by the different colors, and FDR-corrected P values are displayed on the y-axis.

We next sought to understand whether the biofortified Fe diet may influence the genetic capacity of the microbiota, and to characterize the possible functional alterations. Our group recently demonstrated that metagenomic perturbations of the cecal microbiota in chicks influence the severity of a dietary zinc deficiency by, among other pathways, decreasing the capacity of resident bacteria to provide beneficial SCFAs for optimal Zn absorption by the host [38]. The clinical significance of alterations in the metabolic or functional capacity of the host microbiome from the consumption of an Fe biofortified diet has not previously been explored, even though it is clear that the presence of solubilized Fe modulates the gut microbiota. Metagenome functional predictive analysis was carried out using PICRUSt software [39]; OTU abundance was normalized by 16S rRNA gene copy number, identified using the Greengenes database, and Kyoto Encyclopedia of Genes and Genomes (KEGG) orthologue prediction was calculated [39]. In the BFe group, 16 of the 263 (~6%) KEGG metabolic pathways analyzed were differentially-enriched as compared to the SFe group (Fig 5).

All 16 significantly different pathways were depleted in the BFe group when compared to the SFe group. Both the transcription related proteins and mineral absorption KEGG pathways were most significantly depleted in the BFe group (both *P* = 0.004). Fe is an essential metal cofactor for a variety of regulatory transcriptional proteins needed for bacterial viability and bacterial Fe homeostasis, especially in members of the Enterobacteriaceae family [40,41]. Bacteria can sense their environment and alter expression of proteins to increase their viability, such as the expression of high-affinity Fe transporter siderophores [41,42]. The depletion of both the transcription-related proteins and mineral absorption KEGG pathways in the BFe group relative to the SFe group, suggests the presence of Fe-depleted conditions in the BFe gut microbiota, as low concentration of dietary Fe leads to the repression of many bacterial genes involved in Fe uptake [43]. It may also suggest that the luminal Fe in the cecum was not available to the bacteria, or that bacteria which colonized the BFe gut were unable to utilize sufficient Fe, due to the increased concentrations of polyphenols and phytic acid in the biofortified diet, which form Fe complexes. In the gut lumen of SFe animals, due to the poor bioavailability of the dietary Fe, relatively greater Fe-replete conditions caused expansion of bacterial taxa caused expansion of bacterial taxa, such as those belonging to the Enterobacteriaceae family, which have evolved high-affinity Fe transporters. In addition, bacterial pathways responsible for sequestering Fe were upregulated, as observed in the increased expression of pathways belonging to the Genetic Information Processing, Environmental Information Processing, and Cellular Processes KEGG categories. Altogether, the observed metagenomic differences between the groups lends support to the notion that the BFe diet provided less readily-available Fe for the cecal microbiota, and that increased bioavailability in addition to increased levels of Fe-complexing compounds were responsible for these differences.

## Discussion

As is the case in humans and the vast majority of animals, broiler chickens (*Gallus gallus*) harbor a complex and dynamic gut microbiota [44], heavily influenced by host genetics, environment, and diet [45]. There is considerable similarity at the phylum level between the gut microbiota of *Gallus gallus* and humans, with Bacteroidetes, Firmicutes, Proteobacteria, and Actinobacteria representing the four dominant bacterial phyla in both [12,46]. Due to its rapid maturation and well–characterized phenotype during mineral deficiency, *Gallus gallus* has been used extensively as a model of human nutrition, particularly with respect to Fe, and it represents a clinically-validated tool to assess physiological outcomes of low dietary Fe in efficacy trials using Fe biofortified staple food crops [47–53]. In our recent feeding trial published in this journal [11], we demonstrated that a biofortified bean diet (the BFe group in this study) was able to moderately improve Fe status, and that the concurrent increase in the concentration of phytate and polyphenols in these beans likely limited the physiological benefit of increased Fe concentration. Both *in vitro* and *in vivo* arms of this study were conducted under conditions designed to mimic the actual human feeding protocol, and our findings were in concordance with parallel efficacy trials in the target human populations [7,54]. Biofortification is one of several nutritional interventions that aim to alleviate Fe deficiency. These methods—including supplementation, cereal and legume fortification, and complementary feeding—have had some success [52], while additional studies examining how these strategies influence the gut microbiome have helped to improve their safety profile and to guide their further implementation and development [19,22,55]. To date, however, this same scrutiny has not been applied to Fe biofortification of staple food crops. The purpose of this study, therefore, was to address this knowledge gap by exploring the efficacy of biofortification from the perspective of its influence over the gut microbial ecology.

The compositional differences in the microbiota between groups may be explained, in large part, by the increases in phytic acid and in the polyphenols (quercetin and kaempferol) provided by the biofortified diet (S1 and S2 Tables). Members of the Firmicutes are implicated in the metabolism of many phenolic compounds such as isoflavones, flavonols (e.g., quercetin and kaempferol), and flavones [56]. Relative to the SFe group, the BFe group harbored significantly greater levels of members belonging to the Firmicutes, while the Firmicutes phylum was identified by LEfSe as a metagenomic biomarker most likely to explain the physiochemical differences in the BFe group. In addition, the increase in relative abundance of *Faecalibacterium prausnitzii*, a butyrate-producing species belonging to the Firmicutes which was differentially-enriched in the BFe group, has been associated with increased dietary intake of flavonols [57–59]. Further, previous studies of consumption of phenolic-rich foods have shown an increased abundance of taxa that have the capacity for phenolic catabolism [60–62], such as *Enterococcus* spp., *Barnesiella* spp., and members of Dehalobacteriaceae, also differentially-enriched in the BFe group as identified by LEfSe.

Aside from investigating compositional differences that exist between the groups, a major aim of this study was to determine whether ingestion of an Fe biofortified diet would lead to an increased pathogenic bacterial load in the gut microbiota. Dietary Fe supplementation has been associated with an inflammatory-promoting gut microbiota, most likely due to the increased presence of luminal Fe [63], subsequent generation of free radicals, and ensuing epithelial stress and microbial dysbiosis [27]. Many of the nutritional methods used to combat Fe deficiency, such as Fe supplementation and in-home Fe fortification, induce dysbiotic conditions and an expansion of pathogenic bacteria in the gut microbiota of subjects receiving Fe replete diets [19,63]. In contrast to these findings, we did not observe significant changes in α or β bacterial diversity or dysbiosis (as defined by the Firmicutes:Bacteroidetes ratio [64], nor did we find a significant increase in pathogenic taxa in the BFe group that have been previously associated with dietary Fe intake (e.g., *Salmonella* and other Enterobacteria, and *Clostridium spp*.) [19]. In fact, relative to the BFe group, Enterobacteria such as Enterobacteriaceae, were enriched in the SFe group as were bacterial genes responsible for a variety of human diseases. The findings of this study suggest that, in this animal model, Fe biofortification does not seem to adversely affect the composition nor genetic capacity of the gut microbiota. Indeed, the increases in butyrate-producing and other beneficial taxa, such as *Faecalibacterium prausnitzii*, in the BFe group may support the notion that polyphenols, such as quercetin and kaempferol, found in increased concentration in the biofortified diet, favorably modulate the gut microbiome [60]. As it relates to the gut microbiota, and potentially systemically to the host, the increases in polyphenols from biofortification may represent a “side-benefit” as it could confer protection from an outgrowth of pathogenic and opportunistic bacteria. Follow-up studies in at-risk subjects that consume biofortified beans are warranted to assess whether these findings carry over to a human cohort.

In summary, we demonstrate here that a significant remodeling of the gut microbiota occurs in animals receiving a clinically-relevant Fe biofortified diet. Notwithstanding unchanged bacterial diversity, increases in Fe, phytic acid, and/or polyphenols in the biofortified diet influenced the composition of the gut microbiota leading to alterations in its genetic capacity. We suggest that the relative increase in Fe bioavailability of the biofortified diet, in addition to increased levels of Fe bound to phytate and/or polyphenols, selectively modified the gut microbiota by preventing access to luminal Fe by gut microorganisms. Not only was the biofortified diet not associated with an increased dysbiotic or pathogenic microbial load, but, in fact, this group harbored significantly more SCFA-producing and other beneficial bacteria and contained fewer bacterial genes encoding infectious diseases compared to the mildly Fe deficient group. Importantly, these findings are in contrast to other animal and human microbiome studies conducted as part of other interventional studies, such as Fe fortification and Fe supplementation, which have demonstrated improvements in host Fe status at the expense of intestinal dysbiosis, inflammation, and infection. Therefore, under these experimental conditions, the results from this study suggest that Fe biofortification may improve Fe status without negatively altering the structure and function of the gut microbiota. Understanding the effect of Fe biofortification on the gut microbiota may help to further biofortification efforts by improving the safety and efficacy profile of the food crop, as we understand more about the relationship between biofortified diets and the resident gut microbiota. Future investigations should address the interplay between polyphenols, specifically quercetin and kaempferol, and the gut microbiota, especially the role that methanogenic bacteria play in the context of biofortification. Whether the polyphenol profile of biofortified beans can be further modified to support the goals of both gut health and optimal Fe status should be a continuing strategy of biofortification efforts in the context of the microbiome [51,65].

## Conclusion

Nutritional methods of eradicating global Fe deficiency, such as Fe supplementation or Fe fortification, have been moderately efficacious at attaining optimal Fe status. However, any improvement in serum Fe levels comes at the expense of decreased gut health in the form of dysbiosis and infection. This study is the first to report on how Fe biofortification, a clinically-validated method for increasing Fe status, impacts the composition and metagenome of the gut microbiota. Animals (*Gallus gallus*) who consumed the Fe biofortified bean-based diet had less abundance of pathogenic bacteria, with concomitant increases in bacteria that produce short chain fatty acids and have known phenolic catabolic capacity. Collectively, these findings provide evidence that, unlike other nutritional methods of increasing Fe status, Fe biofortification does not appear to increase the pathogenic load in the gut, and they raise the possibility that this strategy can further improve in efficacy and safety as the role of the gut microbiota is explored in additional experimental settings.

## Supporting information

S1 Methods Additional experimental methods and materials. (DOCX)

S1 Table Composition of the experimental diets. (DOCX)

S2 Table Concentrations (μmol/g) of prevalent polyphenols observed in the cream seeded Carioca Bean seed coats. ^a,b^ Within a row, means designated with different letters are significantly different (p < 0.05).
(DOCX)
