# Supplementary material for: Characterizing the gut (Gallus gallus) microbiota following the consumption of an iron biofortified Rwandan cream seeded carioca (Phaseolus Vulgaris L.) bean-based diet
Source: PLoS One. 2017 Aug 10;12(8):e0182431. doi: 10.1371/journal.pone.0182431 (PMC5552115; doi:10.1371/journal.pone.0182431)
Supplement: S1 Methods — (DOCX) [file pone.0182431.s001.docx]

**Characterizing the Gut (*Gallus gallus*) Microbiota Following the Consumption of an Iron Biofortified Rwandan Cream Seeded Carioca (*Phaseolus Vulgaris* L.) Bean-based Diet**

**Spenser Reed,^1,2,#a^ Hadar Neuman,^3^ Raymond P. Glahn,^1^ Omry Koren,^3^ and Elad Tako^1,^***

For the exception of the gut microbiota analysis (#3-#5), all methods listed herein are part of our previously-published study [2].

**1. Animals, Diets, and Experimental Design**

Cornish cross—fertile broiler eggs (*n* = 72) were obtained from a commercial hatchery (Moyer’s chicks, Quakertown, PA). The eggs were incubated under optimal conditions at the Cornell University Animal Science poultry farm incubator. Upon hatching (hatchability rate = 92%), chicks were allocated into two treatment groups on the basis of body weight, gender, and blood hemoglobin concentration (aimed to ensure equal concentration between groups, *n* = 14): 1) Fe Biofortified (BFe): 34.6% cream seeded carioca bean based diet (48.7 ± 1.50 μg Fe/g), and 2) Standard Fe (SFe): 34.6% cream seeded carioca bean based diet (33.7 ± 0.80 μg Fe/g). The two cream-seeded carioca bean lines used in this study were obtained from CIAT (International Center for Tropical Agriculture, Cali, Colombia), and were shipped to Ithaca, New York in sealed containers imported as grain. Upon arrival, all beans were rinsed in ultra-pure (18Ω) water and then cooked using an autoclave for 45 minutes in water and until soft. Beans were then freeze-dried and milled prior to mixing the diets (for all processing, stainless steel appliances were used). Experimental diets (S1 Table) had no supplemental Fe. The specific Rwandese dietary formulation that was used in the study was achieved by a close consultation with and approval of the HarvestPlus nutritionist team, and was based on the menus that were used during the human efficacy trial [[1]](https://paperpile.com/c/gRhdUF/cpBZ). Chicks were housed in a total confinement building (4 chicks per 1 m2 metal cage). The birds were under indoor controlled temperatures and were provided 16 h of light. Each cage was equipped with an automatic nipple drinker and a manual self— feeder. All birds were given ad libitum access to water (Fe content was 0.379 ± 0.012 ppm). Feed intakes were measured daily (from day 1), and Fe intakes were calculated from feed intakes and Fe concentration in the diets.

**2. Blood analysis, hemoglobin (Hb) determination, and tissue collection**

Fe bioavailability was calculated as hemoglobin maintenance efficiency (HME) [[2]](https://paperpile.com/c/gRhdUF/d3syg):

*HME* = $\frac{Hb Fe, mg \left( final \right)- Hb Fe, mg \left( initial \right)}{Total Fe Intake, mg}\times100$

where Hb–Fe (index of Fe absorption) = total body hemoglobin Fe. Hb–Fe was calculated from hemoglobin concentrations and estimates of blood volume based on body weight (a blood volume of 85 mL per kg body weight is assumed) [[2]](https://paperpile.com/c/gRhdUF/d3syg):

*Hb*–*Fe (mg) =*$B.W.\left( kg \right)\times0.085 blood/kg \times Hb (g/L) \times3.35 mg Fe/g Hb$

At study conclusion (day 42) birds were euthanized by CO_2_ exposure. The digestive tracts (colon and small intestine) and liver were rapidly removed from the carcass and separated into various sections for tissue analysis (~1–2 cm; ~2–3 g was taken from small intestine and liver, respectively). The samples were immediately frozen in liquid nitrogen, and then stored in a −80 °C freezer until analysis. All animals were hatched at the same facility. All animal protocols were approved by the Cornell University Institutional Animal Care and Use committee.

**3. Isolation of Total RNA**

Total RNA was extracted from 30 mg of duodenal (proximal duodenum, *n* = 14) and liver tissues (*n* = 14) using Qiagen RNeasy Mini Kit (Qiagen Inc.*,* Valencia, CA, USA) according to the manufacturer’s protocol. All steps were carried out under RNase free conditions. RNA was quantified by absorbance at 260–280 nm. Integrity of the 28S and 18S rRNA was verified by 1.5% agarose gel electrophoresis followed by ethidium bromide staining.

**4. 16S rRNA PCR (Polymerase Chain Reaction) Amplification and Sequencing**

Microbial genomic DNA was extracted from cecal samples using the PowerSoil DNA isolation kit, as described by the manufacturer (MoBio Laboratories Ltd, Carlsbad, CA, USA). Bacterial 16S rRNA gene sequences were PCR-amplified from each sample using the 515F-806R primers for the V4 hypervariable region of the 16S rRNA gene, including 12-base barcodes, as previously described [[3]](https://paperpile.com/c/gRhdUF/TenCy). PCR reactions consisted of 12.5 µL KAPA HiFi HotStart ReadyMix (kit KK2601, Kapa Biosystems, Woburn, MA, USA), 10 µM of each primer, and 10–100 ng DNA template. Reaction conditions consisted of an initial denaturing step for 3 min at 95 °C followed by 31 cycles of [20 s at 98 °C, 15 s at 60 °C and 20 s at 72 °C]. Triplicate PCR reactions were performed for each sample, which were combined and then purified with Ampure magnetic purification beads (Agencourt, Danvers, MA, USA). Purified PCR products were quantified using a Quant–iT PicoGreen dsDNA assay (Invitrogen, Carlsbad, CA, USA). Equimolar ratios of total samples were pooled and sequenced at the Faculty of Medicine of Bar Ilan University (Safed, Israel) using a MiSeq Sequencer (Illumina, Madison, WI, USA).

**5. 16S rRNA Gene Sequence Analysis**

For quality filtering of raw data, sequences with Phred < 20, or shorter than 75% of the expected length were discarded, as well as sequences containing primer mismatches, uncorrectable barcodes, ambiguous bases, or homopolymer runs in excess of 6 bases. The sequences that passed the quality filters were analyzed using the QIIME software package [[4]](https://paperpile.com/c/gRhdUF/TDCpF). Sequences were classified taxonomically using the Greengenes (GG) reference database at a confidence threshold of 80% [[5]](https://paperpile.com/c/gRhdUF/avyuE). The GG taxonomies were used to generate summaries of the taxonomic distributions of OTUs across different levels (phylum, order, family, and genus). To standardize sequence counts across samples with uneven sampling, we randomly selected 22,450 sequences per sample (rarefaction) and used this as a basis to compare abundances of OTUs across samples. For phylogenetic tree-based analyses, each OTU was represented by a single sequence that was aligned using PyNAST [[6]](https://paperpile.com/c/gRhdUF/4j1MT). A phylogenetic tree was built with Fast-Tree [[7]](https://paperpile.com/c/gRhdUF/a6X0J) and used for estimates of α-diversity (within sample diversity, using Faith’s phylogenetic diversity [[8]](https://paperpile.com/c/gRhdUF/iU8Tg)) and β-diversity (between sample diversity, using unweighted and weighted UniFrac [[9]](https://paperpile.com/c/gRhdUF/Or6tJ)). For PD measurements, means and standard errors for given categories were calculated from 100 iterations using a rarefaction of 16,837 sequences per sample. Metagenome functional predictive analysis was carried out using PICRUSt software predictive functional profiling of microbial communities, using 16S rRNA marker gene sequences [[10]](https://paperpile.com/c/gRhdUF/plBFe). Briefly, OTU abundance was normalized by 16S rRNA gene copy number, identified, and compared to a phylogenetic reference tree using the Greengenes database, and was assigned functional traits and abundance based on known genomes and prediction using the Kyoto Encyclopedia of Genes and Genomes (KEGG) [[10]](https://paperpile.com/c/gRhdUF/plBFe).

**6. Polyphenol extraction**

Isolated bean seed coats were prepared by wrapping whole beans in de-ionized water-soaked paper towels until seed coats began to wrinkle and separate from cotyledons. Seed coats were then removed with forceps, dried, and ground to a coarse powder with mortar and pestle. To 1 gram of ground material, 8 mL of methanol: water (50:50 v:v) was added. The slurry was vortexed for 1 minute, placed on an orbital shaker for 25 minutes, then placed in a 30C sonication water bath for 15 minutes, vortexed again for one minute, and centrifuged at 4000 x g for 12 minutes. The supernatant was filtered with a 0.2 μm Teflon syringe filter and stored for later use in a -20C freezer.

**7. Ultra performance liquid chromatography—mass spectrometry (UPLC— MS) analysis of polyphenols**

Seed coat extracts and polyphenol standards were analyzed with an Agilent 1220 Infinity UPLC coupled to an Advion expressionL compact mass spectrometer (CMS). 2 μL samples were injected and passed through an Acquity UPLC BEH Shield RP18 1.7 μm 2.1 x 100 mm column (Waters) at 0.35 mL/min. The column was temperature-controlled at 45°C. The mobile phase consisted of water with 0.1% formic acid (solvent A) and acetonitrile with 0.1% formic acid (solvent B). Polyphenols were eluted using linear gradients of 86.7 to 77.0% A in 0.5 min, 77.0 to 46.0% A in 5.5 min, 46.0 to 0% A in 0.5 min, hold at 0% A for 3.5 min, 0 to 86.7% A in 0.5 min, and hold at 86.7% A for 3.5 min for a total 14 min run time. From the column, flow was directed into a variable wavelength UV detector set at 278 nm. Flow was then directed into the source of an Advion expressionL CMS (Advion Inc., Ithaca, NY), and ESI mass spectrometry was performed in negative ionization mode using selected ion monitoring with a scan time of 50 msec for each of 8 polyphenol masses of interest. Capillary temperature and voltages were 300°C and 100V, respectively. ESI source gas temperature and voltage were 240°C and 2.6kV respectively. Desolvation gas flow was 240 L/hr. LC and CMS instrumentation and data acquisition were controlled by Advion Mass Express software. Identities of polyphenols in bean samples were confirmed by comparison of m/z and LC retention times with authentic standards. Polyphenol quantification was achieved by the use of standard curves and integration of UV absorption peak areas.

**8. Determination of phytic acid concentration in the diet samples**

Dietary phytic acid (phytate)/total phosphorus was measured as phosphorus released by phytase and alkaline phosphatase, following the kit manufacturer’s instructions (*n* = 5, K-PHYT 12/12, Megazyme International, Ireland).

**References**

1. [Haas JD, Luna SV, Lung’aho MG, Wenger MJ, Murray-Kolb LE, Beebe S, et al. Consuming Iron Biofortified Beans Increases Iron Status in Rwandan Women after 128 Days in a Randomized Controlled Feeding Trial. J Nutr. 2016; doi:](http://paperpile.com/b/gRhdUF/cpBZ)[10.3945/jn.115.224741](http://dx.doi.org/10.3945/jn.115.224741)

2. [Tako E, Reed S, Anandaraman A, Beebe SE, Hart JJ, Glahn RP. Studies of Cream Seeded Carioca Beans (Phaseolus vulgaris L.) from a Rwandan Efficacy Trial: In Vitro and In Vivo Screening Tools Reflect Human Studies and Predict Beneficial Results from Iron Biofortified Beans. PLoS One. 2015;10: e0138479.](http://paperpile.com/b/gRhdUF/d3syg)

3. [Caporaso JG, Lauber CL, Walters WA, Berg-Lyons D, Huntley J, Fierer N, et al. Ultra-high-throughput microbial community analysis on the Illumina HiSeq and MiSeq platforms. ISME J. 2012;6: 1621–1624.](http://paperpile.com/b/gRhdUF/TenCy)

4. [Caporaso JG, Kuczynski J, Stombaugh J, Bittinger K, Bushman FD, Costello EK, et al. QIIME allows analysis of high-throughput community sequencing data. Nat Methods. 2010;7: 335–336.](http://paperpile.com/b/gRhdUF/TDCpF)

5. [McDonald D, Price MN, Goodrich J, Nawrocki EP, DeSantis TZ, Probst A, et al. An improved Greengenes taxonomy with explicit ranks for ecological and evolutionary analyses of bacteria and archaea. ISME J. 2012;6: 610–618.](http://paperpile.com/b/gRhdUF/avyuE)

6. [Caporaso JG, Bittinger K, Bushman FD, DeSantis TZ, Andersen GL, Knight R. PyNAST: a flexible tool for aligning sequences to a template alignment. Bioinformatics. 2010;26: 266–267.](http://paperpile.com/b/gRhdUF/4j1MT)

7. [Price MN, Dehal PS, Arkin AP. FastTree: computing large minimum evolution trees with profiles instead of a distance matrix. Mol Biol Evol. 2009;26: 1641–1650.](http://paperpile.com/b/gRhdUF/a6X0J)

8. [Faith DP. Conservation evaluation and phylogenetic diversity. Biol Conserv. 1992;61: 1–10.](http://paperpile.com/b/gRhdUF/iU8Tg)

9. [Lozupone C, Knight R. UniFrac: a new phylogenetic method for comparing microbial communities. Appl Environ Microbiol. 2005;71: 8228–8235.](http://paperpile.com/b/gRhdUF/Or6tJ)

10. [Langille MGI, Zaneveld J, Caporaso JG, McDonald D, Knights D, Reyes JA, et al. Predictive functional profiling of microbial communities using 16S rRNA marker gene sequences. Nat Biotechnol. 2013;31: 814–821.](http://paperpile.com/b/gRhdUF/plBFe)
