# Supplementary material for: Characterizing the gut (Gallus gallus) microbiota following the consumption of an iron biofortified Rwandan cream seeded carioca (Phaseolus Vulgaris L.) bean-based diet
Source: PLoS One. 2017 Aug 10;12(8):e0182431. doi: 10.1371/journal.pone.0182431 (PMC5552115; doi:10.1371/journal.pone.0182431)
Supplement: S1 Table — (DOCX) [file pone.0182431.s002.docx]

| **Ingredient** | **Fe content** | **Standard**  **Bean Diet (SFe)** | **Biofortified**  **Bean Diet (BFe)** |
| --- | --- | --- | --- |
|  | **µg Fe/g, (*n* = 5, by analysis)** | **g/kg (by formulation)** | |
| High-Fe Beans | 106.1±0.204 | – | 346 |
| Low-Fe Beans | 57.10±0.145 | 346 | – |
| Basmati Rice | 0.290±0.006 | 135 | 135 |
| Pasta (non-enriched) | 11.48±0.358 | 70 | 70 |
| Potato flakes | 10.26±0.061 | 215 | 215 |
| Banana Chips | 7.510±0.521 | 115 | 115 |
| Cabbage | 16.32±0.400 | 30 | 30 |
| Tomato powder | 39.92±1.187 | 16 | 16 |
| Orange sweet potatoes | 26.90±0.611 | 73 | 73 |
| Vitamin/mineral premix (no Fe) | 0.00±0.00 | 70 | 70 |
| DL-Methionine | 0.00±0.00 | 2.5 | 2.5 |
| Vegetable oil | 0.00±0.00 | 30 | 30 |
| Choline chloride | 0.00±0.00 | 0.75 | 0.75 |
| Total (g) |  | 1000 | 1000 |
| **Selected components** |  | ***n* = 5 (by analysis)** | |
| Fe concentration (µg/g) | – | 33.7±0.80^b^ | 48.7±1.50^a^ |
| Phytic acid (µg/g) | – | 10605±742^b^ | 13793±1172^a^ |
| Phytate:Fe molar ratio | – | 15.43±0.85^a^ | 10.95±0.65^b^ |
