# Supplementary material for: Characterizing the gut (Gallus gallus) microbiota following the consumption of an iron biofortified Rwandan cream seeded carioca (Phaseolus Vulgaris L.) bean-based diet
Source: PLoS One. 2017 Aug 10;12(8):e0182431. doi: 10.1371/journal.pone.0182431 (PMC5552115; doi:10.1371/journal.pone.0182431)
Supplement: S2 Table — a,b Within a row, means designated with different letters are significantly different (p < 0.05). (DOCX) [file pone.0182431.s003.docx]

| **Compound** | **Biofortified Fe** | **Standard Fe** |
| --- | --- | --- |
| 3,4–dihydroxybenzoic acid | 0.211±0.02^a^ | 0.198±0.002^a^ |
| Catechin | 0.179±0.004^a^ | 0.175±0.020^a^ |
| Quercetin 3–glucoside | 0.085±0.01^a^ | 0.00±0.00^b^ |
| Kaempferol 3–glucoside | 0.302±0.007^a^ | 0.206±0.008^b^ |
| Kaempferol | 0.015±0.001^a^ | 0.015±0.001^a^ |
